# Reply: Reconsidering white matter vulnerability and biomarker interpretation across Lewy body dementia subtypes

**DOI:** 10.1002/alz.70567

**Published:** 2025-08-07

**Authors:** Naomi Hannaway, Angeliki Zarkali, Rohan Bhome, Ivelina Dobreva, George EC Thomas, Elena Veleva, Irene Gorostiaga Belio, Katie Tucker, Amanda Heslegrave, Henrik Zetterberg, Rimona S Weil

**Affiliations:** ^1^ Dementia Research Centre UCL Institute of Neurology London UK; ^2^ Department of Computer Science UCL Hawkes Institute London UK; ^3^ United Kingdom Dementia Research Institute University College London London UK; ^4^ Department of Neurodegenerative Disease UCL Institute of Neurology London UK; ^5^ Department of Psychiatry and Neurochemistry Göteborgs Universitet Mölndal Sweden; ^6^ Department of Neurology Sahlgrenska University Hospital Mölndal Sweden; ^7^ Wellcome Centre for Human Neuroimaging UCL London UK; ^8^ Movement Disorders Centre UCL London UK

1

To the Editor,

We were pleased that Liu and colleagues (2025)^1^ appreciated our recent paper^2^ and found that it provides valuable insights into fluid markers and white matter alterations underlying heterogeneity in Lewy body dementia (LBD).

We agree that macrostructural (fiber cross‐section) and microstructural (fiber density) damage can provide distinct information about the underlying disease processes in LBD. Although macrostructural changes are likely to represent axonal loss or neurodegeneration, microstructural changes are thought to be more sensitive to small vessel disease (SVD).^3^ Both were affected in LBD, but in distinct regions. As Liu and colleagues highlight, differences in SVD remain undetected by current fluid biomarkers but can be quantified through imaging‐derived markers such as white matter hyperintensity (WMH) burden, lacunes, and perivascular spaces. Although Parkinson's disease dementia (PDD) has been shown to be associated with greater WMH^4^ volume, this and other measures of SVD have not yet been directly compared between dementia with Lewy bodies (DLB) and PDD. We did not have available to us the imaging sequences to directly compare measures of SVD, but we agree that this is a valuable direction for future research.

Liu and colleagues^1^ highlight that our work shows that although plasma phosphorylated tau‐217 (p‐tau217; an index of both amyloid beta and tau brain pathology) is increased in LBD, plasma biomarkers cannot currently discriminate between PDD and DLB. However, combining plasma p‐tau217 and other fluid markers with advanced imaging techniques, such as fixel‐based analysis, enabled us to shed light on underlying tissue changes and to better understand heterogeneity in LBD. As we describe,^2^ standard diffusion tensor imaging (DTI) metrics also showed changes in diffusivity in LBD relative to cognitively unimpaired people with Parkinson's disease who were at low‐risk for dementia (PD), but could not detect differences between PDD and DLB, as they are not sensitive to changes in white matter micro‐ and macro‐structure. Likewise, studies comparing gray matter atrophy between PDD and DLB have found inconsistent results and similarly do not robustly distinguish between DLB and PDD^5^, ^6^, ^7^.

We have also recently shown that, instead, other sequences with sensitivity to tissue composition show differences between PDD and DLB. We used quantitative susceptibility mapping (QSM), a neuroimaging technique sensitive to brain tissue iron concentrations, and found altered QSM in widespread regions in PDD compared to DLB, as well as an association between QSM and overall disease severity.^8^

We concur with Liu and colleagues that future research could further advance this work by examining longitudinal trajectories with combined fluid markers and advanced imaging techniques. For example, we have shown in people with PD without cognitive involvement that fixel‐based analysis and QSM each have sensitivity to predict future cognitive decline and other poor outcomes.^9^, ^10^ These and other quantitative imaging techniques are likely to be important in the longitudinal evaluation of LBD, both to predict future decline and to monitor disease progression.

These multimodal approaches, using combinations of imaging and fluid biomarkers, will advance our understanding of the heterogeneity of LBD and even shed light on how it progresses over time. This will have important implications for clinical practice and potentially enable targeted approaches for the treatment of LBD in the future.

## CONFLICT OF INTEREST STATEMENT

R.S.W. has received speaking and writing honoraria from GE Healthcare, Bial, Omnix Pharma, and Britannia; and consultancy fees from Therakind and Accenture. All other authors report no conflicting interests. Any author disclosures are available in the Supporting Information.

## Supporting information



Supporting Information
